# Plant phenological asynchrony and community structure of gall‐inducing insects associated with a tropical tree species

**DOI:** 10.1002/ece3.4477

**Published:** 2018-11-01

**Authors:** Marcilio Fagundes, Renata Cristiane Ferreira Xavier, Maurício Lopes Faria, Laura Giovanna Oliveira Lopes, Pablo Cuevas‐Reyes, Ronaldo Reis‐Junior

**Affiliations:** ^1^ Departamento de Biologia Geral CCBS Programa de Pós‐Graduação em Biodiversidade e Uso dos Recursos Naturais Universidade Estadual de Montes Claros Montes Claros Minas Gerais Brazil; ^2^ Laboratorio de Ecología de Interacciones Bióticas Facultad de Biología Universidad Michoacana de San Nicolás de Hidalgo Morelia Michoacán México

**Keywords:** community structure, gall‐inducing insect diversity, interspecific competition, leaf flushing, null models, plant–herbivore synchronism

## Abstract

The dynamics of occurrence of target organs in plant populations produces windows of opportunity that directly and indirectly affect the structure of herbivore communities. However, mechanisms that drive herbivore specialization between resource patches are still poorly known. In this study, we tested three hypotheses related to variation in host plant phenology and community structure (i.e., composition, richness, and abundance) of gall‐forming species: (a) plants with early leaf‐flushing in the season will have greater vegetative growth and high contents of secondary chemical compounds; (b) gall‐inducing insect community structure changes among temporary resource patches of the host; and (c) interspecific competition is a probable mechanism that drives gall‐inducing insect community structure on *Copaifera langsdorffii*. We monitored daily a total of 102 individuals of the super‐host *C. langsdorffii* from August 2012 to May 2013, to characterize the leaf flushing time of each host plant. The leaf flushing time had a positive relationship with the number of folioles per branch and a negative relationship with branch growth. We sampled a total of 4,906 galls belonging to 24 gall‐inducing insect species from 102 individuals of *C. langsdorffii*. In spite of some gall‐inducing species presented high abundance on early leaf‐flushing plants, direct and indirect effects of plant phenology on galling insect abundance was species dependent. At the community level, our study revealed that the quality and quantity of plant resources did not affect the richness and abundance of gall‐inducing insects associated with *C. langsdorffii*. However, the richness and composition of gall‐inducing species varied according to the variation in leaf flushing time of the host plant. The results of null model analysis showed that galls co‐occurrence on *C. langsdorffii* trees differ more than expected by chance and that interspecific competition can be one potential mechanism structuring this gall‐inducing insect community.

## 1 INTRODUCTION

The resources obtained by plants are allocated to different functions such as growth, reproduction, and production of chemical compounds associated with defence against herbivorous insects (Costa, Reis‐Júnior, Queiroz, Maia, & Fagundes, 2016; Herms & Mattson, 1992; Stamp, 2003). Variation in leaf flushing time within a plant population can play a relevant role in plant–herbivore interactions (Fagundes, 2014; Forister, 2005). Directly, plant phenology can affect herbivore actions using the host satiation mechanism or producing resource patches that vary over time (Aide, 1993; Fei, Gols, & Harvey, 2014; Souza & Fagundes, 2017; Van Schaik, Terborgh, & Wright, 1993). Indirectly, variation in leaf flushing time produces resource patches that change in quality for herbivores. For example, in seasonal environments, plants with early leaf‐flushing can accumulate more resources during an extended vegetative stage, resulting in both a greater vegetative growth and accumulation of secondary compounds, which in turn, affect herbivores performance (Costa et al., 2016; Fagundes et al., 2016; Forister, 2005; Yukawa, 2000). Thus, intrapopulational variation in leaf plant phenology can affect plant quality, preference, and incidence of herbivorous insects on plants (Dahlgren, Zeipel, & Ehrlén, 2007; Nord, Shea, & Lynch, 2011; Parachnowitsch, Caruso, Campbell, & Kessler, 2012).

Several studies show that interplant phenological variation affects spatial and temporal distribution of herbivorous insects (Lewinsohn, Novotny, & Basset, 2005; Novotny et al., 2006; Thompson & Gilbert, 2014) and plays an important role in plant–herbivore coevolution (Fei et al., 2014; Johansson, Kristensen, Nilsson, & Jonzén, 2015). Herbivorous such as gall‐inducing insects have their life cycle synchronized with the production of host plant organs because they require undifferentiated tissues to initiate gall induction (Maldonado‐López, Cuevas‐Reyes, Stone, Nieves‐Aldrey, & Oyama, 2015; Weis, Walton, & Greco, 1988; Yukawa, 2000). Consequently, the timing and site of oviposition are crucial to successful gall development in many gall‐inducing insects (Hayward & Stone, 2005; Stone, Schönrogge, Atkinson, Bellido, & Pujade‐Villar, 2002), representing a “window of opportunity” because gall‐inducing insects attack the first leaf flush of their host plants (Hayward & Stone, 2005; Yukawa, 2000). In this case, the high specialization of gall‐inducing insects on their host plants confers them high‐quality resources (Thompson & Gilbert, 2014; Yukawa & Akimoto, 2006). Therefore, plants are under natural pressures to reduce the action of herbivores using the host satiation mechanism, by synchronizing their phenological events, or producing resource patches that vary in quality (e.g., concentrations of secondary metabolites) and quantity (e.g., number of leaves) over time (Fei et al., 2014; Forister, 2005; Parachnowitsch et al., 2012; Souza & Fagundes, 2017; Van Schaik et al., 1993).

Temporal dynamics of occurrence of target organs in an asynchronous plant population produce windows of vulnerability in which different herbivore species can specialize (Fei et al., 2014; Fox, Waddell, Groeters, & Mousseau, 1997; Yukawa, 2000). In fact, plants with early‐leaf flushing are usually more attacked by sessile herbivores such as gall‐inducing insects, because those plants provide more resources and longer time for herbivore development (Forister, 2005; Yukawa & Akimoto, 2006). In contrast, herbivores with a short larval phase can better adapt to plants with late leaf flushing (Fagundes et al., 2016; Singer & Parmesan, 2010). Different races of the same herbivore species can use temporary resource patches in different ways (Mopper, 2005). These findings suggest that specialization of gall‐inducing insects on temporary resource patches can shape the structure of herbivore communities (i.e., composition, richness, and abundance of species) associated with a host plant species (Edmunds & Alstad, 1978; Egan & Ott, 2007; Mopper, 2005; Oliveira, Mendonça, Moreira, Lemos‐Filho, & Isaias, 2013; Yukawa & Akimoto, 2006). Competition (Kaplan & Denno, 2007; Mopper, 2005), escape from natural enemies (Egan & Ott, 2007; Karban & Agrawal, 2002), and resource depletion (Revilla, Encinas‐Viso, & Loreau, 2014) are possible biological factors used to explain the variation in the herbivore community structure among temporary resource patches of the host plant. However, the role of those biological factors in shaping the structure of natural communities has been little tested experimentally (see Gurevitch, Morrison, & Hedges, 2000; Oliveira et al., 2013; Pereira et al., 2014).

Gall‐inducing insects exhibit at least three characteristics that make them excellent models to access the importance of interactions within and between different trophic levels in the organization of herbivore communities. First, the adult life time of specialized gall‐inducing insects is relatively short and gall‐inducing insects are extremely specialized on the host plant taxon and tissue exploited (Carneiro et al., 2009; Fagundes, Neves, & Fernandes, 2005). This characteristic leads to an intimate temporal relationship between the life cycle of gall‐inducing insects and the occurrence of the target organ in the plant. Second, the galls are sessile and their formation is associated with an alteration in plant resource allocation, which occurs a few hours after oviposition (Höglund, 2014), with possible effects on the oviposition site selection by females of other galling insects (Cornelissen, Guimarães, Viana, & Silva, 2013). Third, the success of the gall‐inducing insect also depends on the selection of the most vigorous organ of the plant (resource quality) and the number of oviposition sites (resource abundance) available for its development (Höglund, 2014; Price, 1991). Moreover, the performance of gall‐inducing insects is also affected by the interaction between plant phenology and top–down forces (Fagundes et al., 2005; Hood & Ott, 2010; Johansson et al., 2015) as well as by biological interactions that occur within the same trophic levels (Cornelissen et al., 2013; Johansson et al., 2015).

Previous studies have shown that competition between endophagous insects as leaf miners and gall‐inducing insects can arise when sites for oviposition and insect development are a limiting resource (Cornelissen et al., 2013). When experimental manipulations are impossible due to the nature of the study system, the comparison of the observed patterns of species occurrence with patterns that might be expected to occur by chance alone is a conventional approach to study competition between species (Cornelissen & Stiling, 2008; Morin, 2011). Many authors have used analysis of co‐occurrences to show the role of competition on community organization of sedentary insects on host plant (e.g., Cornelissen et al., 2013; Kaplan & Denno, 2007; Tack, Ovaskainen, Harrison, & Roslin, 2009). *Copaifera langsdorffii* (Fabaceae) is a super‐host species for gall‐inducing insects (Sensu Veldtman & McGeoch, 2003) and exhibits substantial interplant variation in the timing of the leaf flushing (Fagundes, 2014). We used the system *Copaifera langsdorffii*–galling insects to test the following hypotheses: (a) plants with early leaf‐flushing in the season will have greater vegetative growth and high concentrations of secondary chemical compounds; (b) gall‐inducing community structure changes among temporary resource patches of the host and (c) the actual pattern of gall co‐occurrence on host plants differ from that expected by chance.

## 2 MATERIAL AND METHODS

### 2.1 Study area

This study was carried out in a private reserve with an area of approximately 25 ha, located in Montes Claros, northern Minas Gerais, Brazil (16^o^40′26″S and 43^o^48′44″W). The soil of the study area is dystrophic and covered by trees and shrubs with distorted branches and thick bark, with height between 2 and 10 m (Souza, Solar, & Fagundes, 2015). The region is located in the transition between the biomes Cerrado and Caatinga of Brazil. Its climate is semiarid with a dry season from March to September and a rainy season from October to February. The average annual temperature is 23°C, and the rainfall is approximately 1,000 mm/year, mainly concentrated from November to January (Costa et al., 2016).

### 2.2 The system

*Copaifera langsdorffii* Desf. (Fabaceae) is a tropical tree species that reaches up to 20 m in height in the Brazilian Cerrado. In the study area, these trees show complete deciduousness in the dry season (mainly from July to September) and leaf flushing occurs right after the fall of old leaves produced in the previous year. Flowering occurs from November to December and fruits ripe from August to September (Fagundes et al., 2013). Fruiting set is supra‐annual, that is, there are years of intense fruiting set followed by years of low or no fruit production (Souza & Fagundes, 2017). *C. langsdorffii* has the most diverse fauna of gall‐inducing insects in the Neotropics (24 species) and a broad diversity of free‐living herbivore insects (Costa et al., 2016).

### 2.3 Data collection

In March 2012, we selected and marked 102 individual adults of *C. langsdorffii* with a height that ranged between 8 and 12 m, abundant crown, and absence of lianas and parasitic plants. *C. langsdorffii* trees showed irregular distribution in the study area and the distance between trees varied from 7 to 45 m. We daily monitored all trees selected for the study, from August 2012 to May 2013 to determine the period of new leaf flushing of each individual plant. In June 2013 (prior to leaf fall), we collected ten terminal branches (approximately 30 cm long) from each tree and in this way, we were able determine the relative temporal variation (days) in leaf flushing among individual trees in the population. We collected those branches from different parts of the tree crown, to obtain samples from the whole individual and avoid a bias caused by the effect of microhabitat on branch development (Costa, Fagundes, & Neves, 2010). We used those samples to quantify branch growth (mean internodes length), average number of folioles per branch, leaf phenol concentration, and intensity of attack by gall‐inducing insects. We determined the growth of each branch by dividing the length of the main branch by its number of nodes. We identified the galls present in those branches following Costa et al. (2010).

### 2.4 Phenol quantification

The leaf phenol content was determined with spectrophotometry using the Folin–Ciocalteu methodology (Swain & Hillis, 1959), and which included gallic acid as standard. Initially, leaf extract was prepared for each individual plant by diluting 0.5 g grinded leaves in one mL of methanol. The 0.5 g of leaves was obtained from 50 mature leaves collected from those ten branches in each plant according to the method mentioned above. Next, 500 μl of the extract was transferred to tubes containing 250 μl of Folin–Ciocalteu's reagent. After waiting for ten minutes, 500 μl of a sodium carbonate solution (10% w/v) was added to the samples. The tubes were then allowed to stand at room temperature for 30 min before absorbance was measured at 743 nm. The concentration of polyphenol in the samples was derived from a standard curve of gallic acid ranging from 20, 30, 40, 60, and 80 μg/ml. Total phenolic content was expressed as gallic acid equivalents (GAE) in mg/g of dry leaf extracts.

### 2.5 Statistical analysis

Generalized linear models (GLMs) were used to assess the effects of the variation in plant flushing time on branch growth, average number of folioles, and leaf phenol contents. The branch growth, the average number of folioles, or the leaf phenol content were considered as the response variables, and the relative variation in leaf flushing time among plants was considered the explanatory variable. Considering that the response variables are continuous, we tested the models using a *F* test based on the Gaussian distribution. After those tests, the models were submitted to analysis of residuals to test their adequacy to statistical assumptions (Crawley, 2007).

To determine the effects of the variation in leaf flushing time, branch growth, leaf phenol contents, and average number of folioles per branch on the average abundance of each gall species, total richness, and mean of total abundance of galls (i.e., sum of all galls divided by number of branches sampled per plant), we performed generalized linear models (GLMs). In model constructions, the abundance of each gall‐inducing insects as well as total richness and average abundance of gall‐inducing were used as response variables. The explanatory variables were leaf phenol contents, branch growth, average number of folioles, leaf‐flushing time, and the interaction among all explanatory variables. However, these tests were done only for gall‐inducing insect species that were present at least in 25% of all sampled plants to avoid that high number of zero values mask the result of statistical analysis. We tested the models related to gall‐inducing insect richness with a chi‐squared test based on the Poisson distribution as they contained count data. To test the models of average abundance, we used a F test based on the Gaussian distribution, as they contained continuous data (Crawley, 2007). To control for the effect of the entry sequence of explanatory variables used in the models, we use a stepwise model selection analysis in the package MuMIn for R (Bartón, 2015). In this analysis, the selection of the most parsimonious model is based on the values of the Akaike Information Criterion (AIC). Finally, we submitted the most parsimonious model to an analysis of residues to test for the adequacy of the model to statistical assumptions (Crawley, 2007). In all analyses, we tested for the presence of influent points that could interfere with the results. We removed those influent points from significant models and ran the analyses again. All analyses were run in the program R (R Core Team, 2015).

To test whether the community composition of gall‐inducing insects varied among plants with early, intermediate, and late sprouting, we used a nonmetric multidimensional scaling analysis followed by an Anosim permutation test. We divided the plants into early (plants that flushed until the 15th day, *n* = 40), intermediate (plants that flushed from the 17th to the 43rd day, *n* = 29), and late‐flushing plants (plants that flushed after the 46th day, *n* = 33). The Jaccard similarity index calculated based on the matrix of presence/absence of gall species was used in the above mentioned tests. All analyses were conducted in the R software (R Core Team, 2015).

We used null models to compare the observed and random patterns of gall‐inducing insect occurrence in all plants of the population. A C‐score index (Stone & Roberts, 1990), calculated based on the matrices of presence/absence of galls per plant, was used to quantify the co‐occurrence of galls. The null hypothesis was that the presence of one gall species in the plant does not influence the occurrence of other gall species in the same plant. Hence, when the value of the co‐occurrence index calculated for the original matrix (observed C‐score) was outside 95% of the values found in the distributions of randomized C‐scores, the null hypothesis must be rejected. In that case, we infer that the distribution of gall‐inducing species on plants is determined by interspecific biological interactions (Ribas & Schoereder, 2002). To test for variation in the distribution of observed and expected matrices, we used fixed–fixed models with 5,000 randomizations. All analyses were conducted in the R software (R Core Team, 2015).

## 3 RESULTS

**Figure 1:**
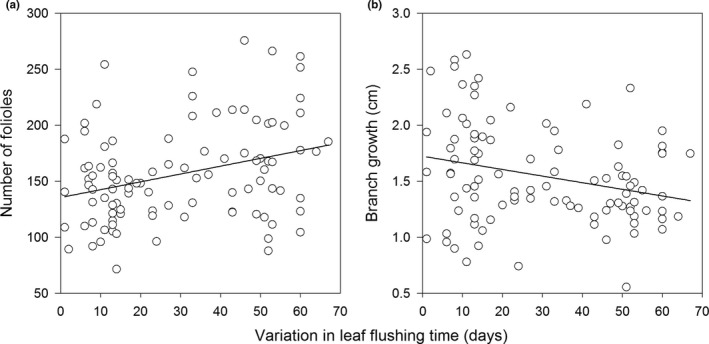
Relationship between variation in leaf flushing time and number of folioles per branch (a) and branch growth (b) of *Copaifera langsdorffii* plants

Leaf‐flushing time in the studied population started on July 27, 2012, and continued until October 01, 2012. Hence, interplant variation in leaf‐flushing time of *C. langsdorffii* lasted 67 days. The leaf‐flushing time had a positive relationship with the number of folioles per branch (*F* = 10.755, *p *=* *0.001; Figure 1a) and a negative relationship with branch growth (*F* = 7.429, *p *=* *0.008; Figure 1b). However, leaf phenol contents did not vary as a function of leaf flushing time in *C. langsdorffii* plants (*F* = 0.054, *p *=* *0.817).

**Figure 2:**
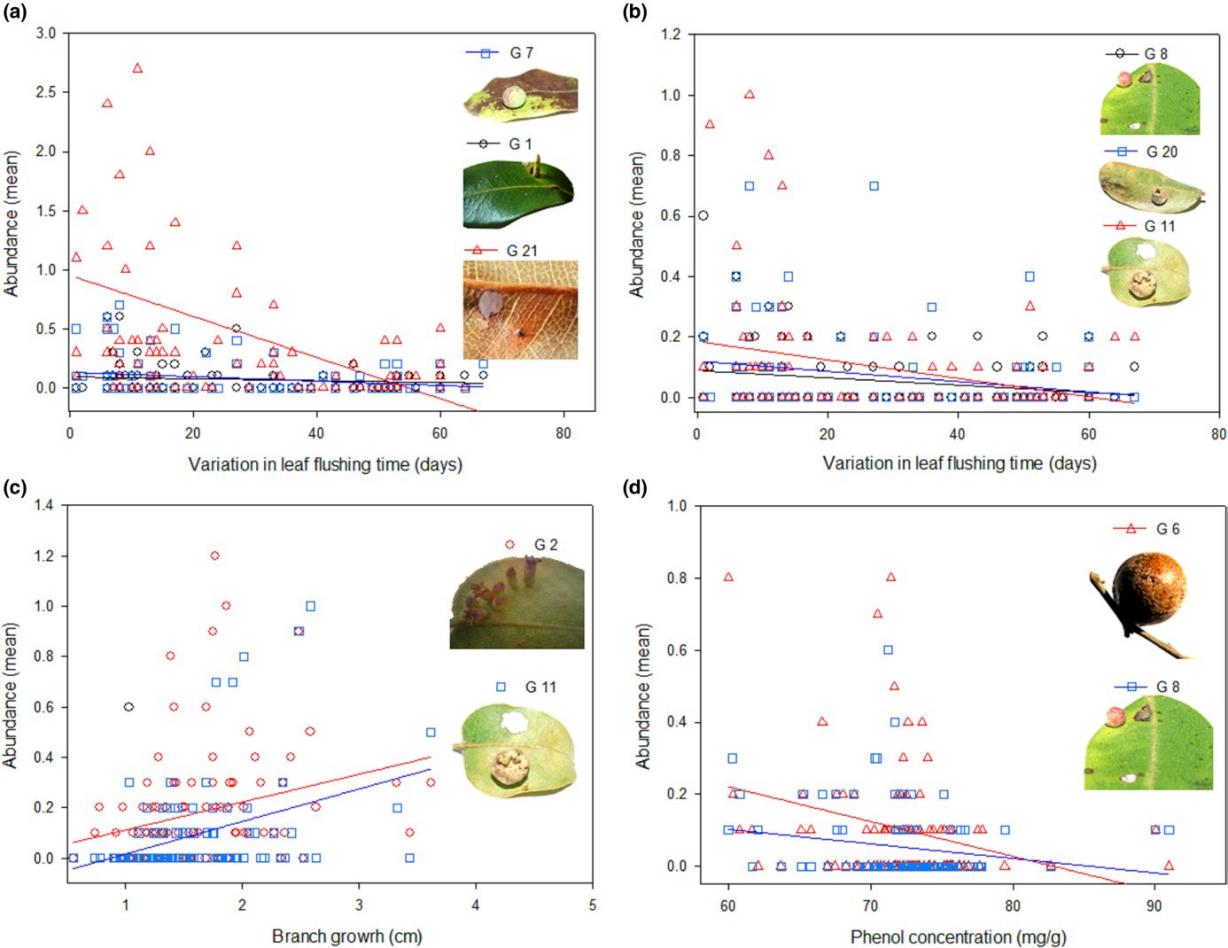
Relationship between gall‐inducing insect abundance and variation in leaf flushing time (a,b), branch growth (c), and leaf phenol contents (d) of *Copaifera langsdorffii* plants

**Figure 3:**
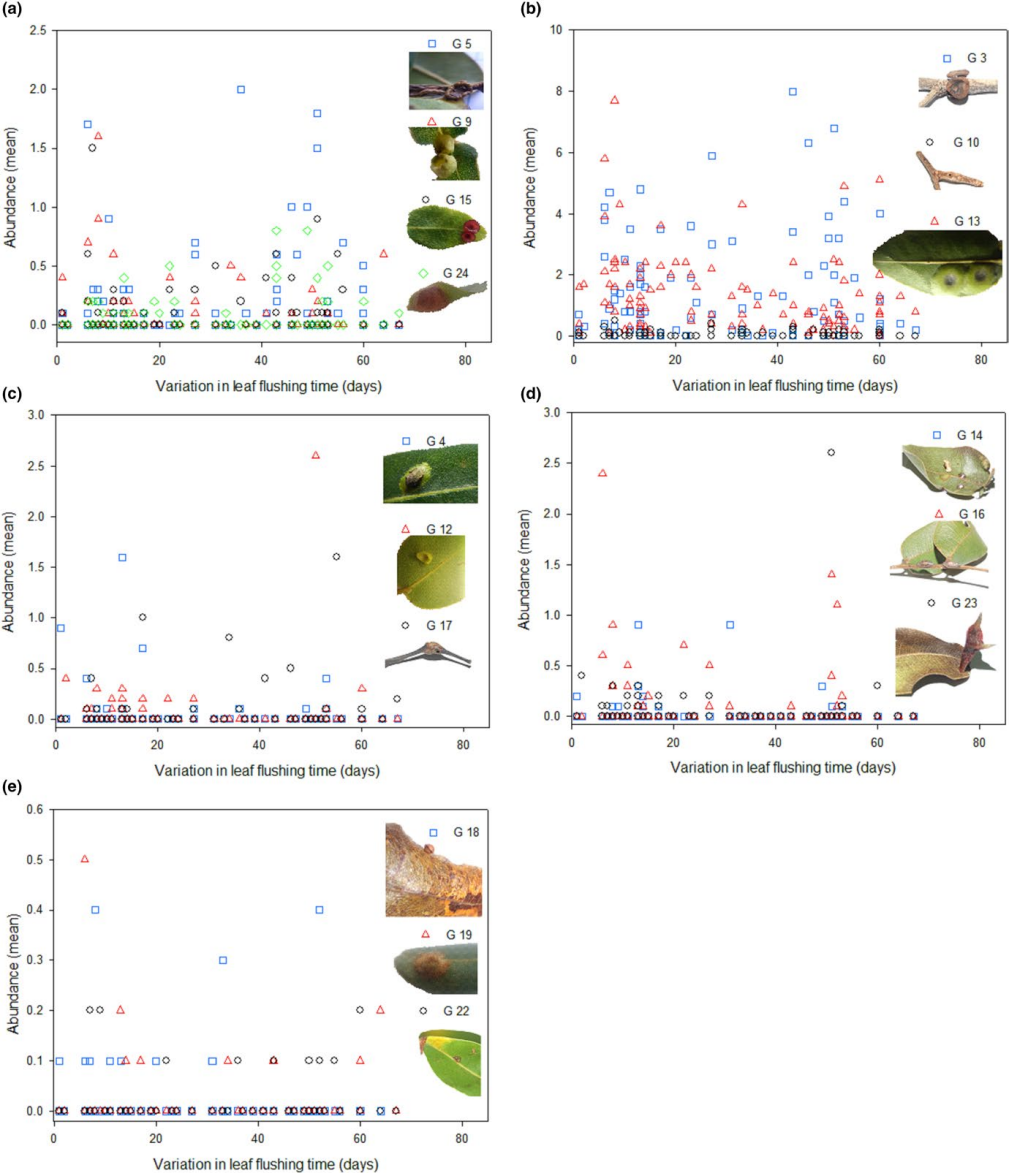
Abundance of gall‐inducing insect associated with *Copaifera langsdorffii* plants. Gall‐inducing insects shown in figure (a,b) occur in high frequency on host plant (>25% of plants) but were statistically not affected by tested explanatory variables. Gall‐inducing insects shown in figure (c–e) occur in low frequency (<25% of plants) on *Copaifera langsdorffii* plants

We sampled a total of 4,906 galls belonging to 24 gall‐inducing insect species from 102 individuals of *C. langsdorffii*. Most gall‐inducing species (15 species) were present in more than 25% of the 102 sampled plants. Among these 15 gall‐inducing insects, the abundance of only eight species were statistically affected by explanatory variables (Table 1). In fact, the abundance of six species was negatively related with leaf flushing time (Figure 2a,b), two gall‐inducing species were positively related with branch growth (Figure 2c), two gall‐inducing insects were negatively related with leaf phenol content (Figure 2d), and seven gall‐inducing insects were not statistically affected by explanatory variables (Figure 3a,b). A total of nine gall‐inducing species presented low frequency on sampled plants (<25%) and was not statistically analyzed (Figure 3c–e).

**Table 1:** Minimum adequate models showing the effects of the explanatory variables (variation in leaf flushing time, branch growth, and leaf phenol contents) on the abundance of gall‐inducing species associated with *Copaifera langsdorffii*

Response variables	Explanatory variables	Error distribution	Deviance	Residual deviance	*F*	*p*
G1 abundance	Leaf flushing	Gaussian	0.0084	1.2391	6.6817	0.0114
G2 abundance	Branch growth	Gaussian	0.3587	5.1589	6.8839	0.0101
G6 abundance	Phenol	Gaussian	0.2122	3.9274	5.1861	0.0249
G7 abundance	Leaf flushing	Gaussian	0.1359	2.1417	6.284	0.0143
G8 abundance	Leaf flushing	Gaussian	0.0578	0.9641	5.8722	0.0174
	Phenol		0.0402	0.9239	4.0886	0.0460
G11 abundance	Leaf flushing	Gaussian	0.3106	3.2473	9.9582	0.0021
	Branch growth		0.2219	3.0254	7.1162	0.0089
G20 abundance	Leaf flushing	Gaussian	0.1127	1.8782	5.9379	0.0166
G21 abundance	Leaf flushing	Gaussian	11.837	121.70	9.629	0.0025

**Figure 4:**
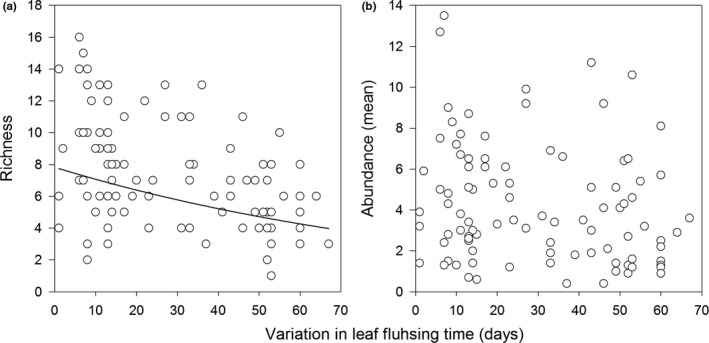
Relationship between variation in leaf flushing time and richness (a) and abundance (b) of all gall‐inducing insects associated with *Copaifera langsdorffii* plants

Among the explanatory variables tested, only leaf flushing time affected total gall richness (Deviance = 20.548, *p *<* *0.001). We observed a negative relationship between total gall‐inducing insect richness and leaf flushing time, which suggests that early leaf‐flushing plants are more specious (Figure 4a). No explanatory variable affected total abundance of galls associated with *C. langsdorffii* plants (Figure 4b).

**Figure 5:**
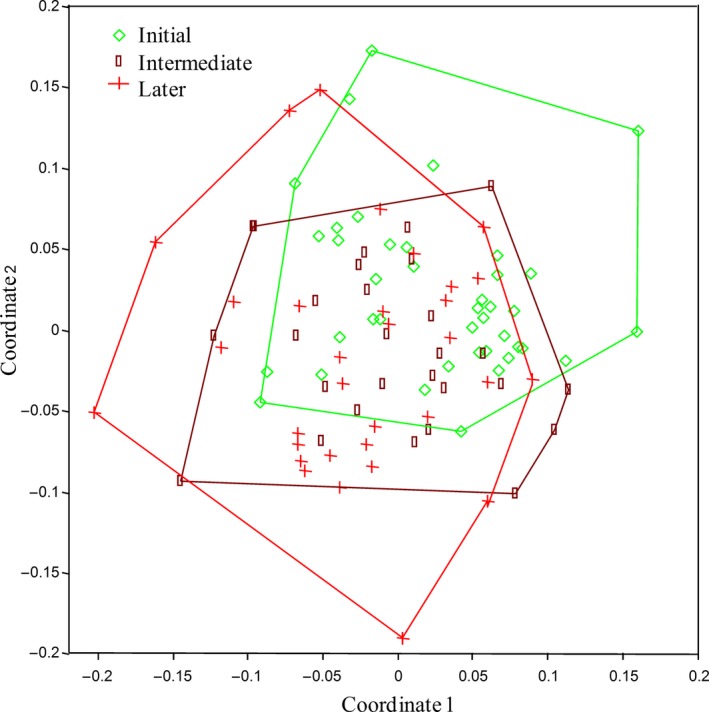
Variation in the composition of gall‐inducing insects between *Copaifera langsdorffii* plants with early, intermediate, and late leaf flushing. The composition of the gall‐inducing insects community varied between plants with early and late leaf flushing (Anosim: *p *=* *0.0034). There is a tendency of change in the gall‐inducing insects community between plants with early and intermediate leaf flushing (Anosim, *p *=* *0.054). The composition of the gall‐inducing insect community did not vary between plants with intermediate and late leaf flushing (Anosim: *p *=* *0.98)

The composition of the gall community varied among plant phenological classes (Anosim: *p *=* *0.018, Figure 5). We observed the formation of two plant groups: the first composed by galls present in plants with early leaf phenology and the second composed by galls of plants with intermediate and late leaf phenology.

**Figure 6:**
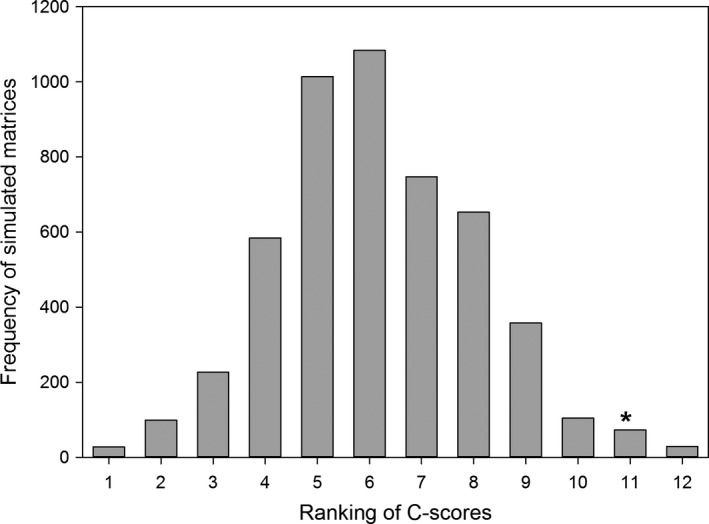
Frequency of simulated C‐scores, using fixed‐fixed model, of gall‐inducing insects associated with 102 plants of *Copaifera langsdorffii*. The asterisk indicates where the observed C‐scores coincided with the frequency class of the C‐scores of the 5,000 simulated matrices (C‐score observed = 230.43, mean of simulated indices = 228.06, minimal value of simulated indices = 225.06, maximal value of simulated indices = 231.44)

The observed values of the C‐score index did not fall within the 95% limits of frequency distribution of the 5,000 randomized matrices (*p *=* *0.019, Figure 6). Thus, the null hypothesis was rejected and the hypothesis of biological mechanisms conditioning the gall‐inducing insect occurrence on *C. langsdorffii* must be accepted.

## 4 DISCUSSION

Plants with early leaf‐flushing in the season usually show great vegetative development and accumulate greater amounts of secondary compounds (Ehrlen & Munzbergová, 2009; Parachnowitsch et al., 2012), but trade‐offs in resource allocation among different drains is a common phenomenon in plants that affect their development (Nord et al., 2011). For example, when the resources are scarce to plants (i.e., water and nutrients), they show greater growth in branches but less in vegetative biomass (Costa et al., 2016). Our results showed that plants with early leaf‐flushing had greater shoot growth with a smaller number of folioles. Hence, there seems to be a trade‐off in resource allocation between branch growth and the number of leaves produced by *C. langsdorffii* as a result of the variations in plant leaf‐flushing time. However, no relationship was observed between leaf phenol content and the variation in leaf flushing time, suggesting that resource allocation among different functions of the plants is a complex phenomenon that still deserves more attention (Costa et al., 2016; Sandvik & Eide, 2009). In our case, considering that *C. langsdorffii* trees are deciduous is possible that the replacement of all leaves annually can mask the possible relationship between leaf phenol content and the variation in leaf flushing time. Moreover, it is important to point out that methodology used to quantify leaf phenol concentration can produce bias that affects final interpretation of results (see Appel, Govenor, D'Ascenzo, Siska, & Schultz, 2001).

Phenological asynchrony in plant populations can affect plant–herbivore interactions directly by producing patches of temporally unpredictable resources (Fei et al., 2014; Souza & Fagundes, 2017) and indirectly by producing resources that vary in quality and quantity over time (Forister, 2005; Nord et al., 2011; Parachnowitsch et al., 2012). In a population perspective, early leaf‐flushing plants usually produce more vigorous growth modules (Fox et al., 1997; Vitou, Skuhravá, Skuhravý, Scott, & Sheppard, 2008; Yukawa, 2000), which are preferentially attacked by herbivores (Price, 1991). In our study, of the 15 more frequent gall‐inducing insect species that were statistically analyzed, eight gall species were directly affected by plant phenology or indirectly by plant quality (see Figure 2a–d) Therefore, the abundance of seven gall‐inducing species (46.47%) was not statistically affected directly or indirectly by host plant phenology. Thus, others factors may be operating at the population or community level affecting the abundance and distribution of gall‐inducing insects associated with *C. langsdorffii* (Maldonado‐López et al., 2015).

At the community level, our study revealed that the quality (branch size and leaf phenol contents) and quantity (number of folioles) of plant resources did not statistically affect the richness and abundance of gall‐inducing insects associated with *C. langsdorffii*. Moreover, our results showed that although overall gall abundance was not affected by any of the explanatory variables tested, the richness, and composition of gall species varied according to the variation in leaf flushing time of the host plant. At least, three important points can emerge from these results. First, gall community structure seems to be shaped directly by plant phenology and not by the variations of resource quantity or quality over time. Second, because total gall abundance does not change in function of variation in leaf flushing of host plant, the resource (i.e., sites for adult oviposition) can be exploited evenly throughout time by the gall community associated with the host *C. langsdorffii*. Third, the variation in gall composition suggests a temporal variation in resource use among different species of gall‐forming insects associated with *C. langsdorffii*.

Because gall‐inducing insect species have the ability to manipulate the growth and development of plant tissues (Cuevas‐Reyes, Quesada, Siebe, & Oyama, 2004; Cuevas‐Reyes, Siebe, Martínez‐Ramos, & Oyama, 2003) and may also be capable of modifying host nutritional quality and plant secondary metabolites for protection against natural enemies (Pascual‐Alvarado, Cuevas‐Reyes, Quesada, & Oyama, 2008), this insect guild tends to be less affected by quality of host plant tissues (Höglund, 2014). Therefore, adult females must select the most adequate sites for oviposition for their offspring to reach higher success (Cuevas‐Reyes, Quesada, Hanson, Dirzo, & Oyama, 2004). For example, galling insects generally oviposit on buds that give rise to more vigorous branches to ensure greater offspring performance (Faria & Fernandes, 2001; Price, 1991). Thus, the dispute for more adequate sites for oviposition is a possible factor that drives the organization of gall‐inducing insect communities especially in super‐host plants (Reitz & Trumble, 2002). This idea is in agreement with our results, because the richness and composition of galling insects were not affected by quality of the host plant, but these two aspects of the community changed as a function of leaf flushing time of the host plants.

In spite of many studies showing that the galling community structure can be affected by plant phenology (e.g., Mopper, 2005; Oliveira, Pallini, & Janssen, 2016), the mechanisms that drive this pattern of gall occurrence within host plant populations are still poorly understood. The intimate temporal relationship between the life cycle of gall‐inducing insects and the occurrence of the target organ in an asynchronic host plant population can intensify the dispute for sites for oviposition, generating a pattern of temporal repulse among galling insects. Recent studies using analysis of co‐occurrence have shown that interspecific competition is a mechanism capable of shaping the community structure of sessile herbivores (e.g., Cornelissen & Stiling, 2008; Cornelissen et al., 2013; Tack et al., 2009). The results of the null model analysis showed that the values of co‐occurrence indices of galls of the observed matrices were greater than the co‐occurrence indices of simulated matrices. Hence, our null models analysis suggests that co‐occurrence of galls on *C. langsdorffii* trees differ more than expected by chance and interspecific competition can be one potential mechanism structuring this gall community.

In summary, our study showed that *C. langsdorffii* is a deciduous tree species with broad intrapopulation phenological asynchrony in leaf flushing time. In spite of some gall species presenting high abundance on early‐flushing plants, direct and indirect effects of plant phenology on galling insect abundance was species dependent. Finally, the structure of galling insect community associated with *C. langsdorffii* change in function of leaf flushing time of host plant and interspecific competition seem be responsible by these gall community organization.

## AUTHOR CONTRIBUTIONS

MF, MLF, PCR originally formulated the idea. MF, MLF, RRJ developed methodology. MF, RCFX, LGOL conducted fieldwork. MF and RRJ performed statistical analyses. LGOL, MF, RCFX, and PCR wrote the manuscript. LGOL, RCFX, and MF formatted and submitted the manuscript.

## CONFLICT OF INTEREST

None declared.

## DATA ACCESSIBILITY

The data supporting this study and all analyses are available at doi: 10.5061/dryad.fk0v0fq.
